# Therapeutic Potential of Hydrogen Sulfide in Ischemia and Reperfusion Injury

**DOI:** 10.3390/biom14070740

**Published:** 2024-06-22

**Authors:** Xutao Sun, Siyu Wu, Caiyun Mao, Ying Qu, Zihang Xu, Ying Xie, Deyou Jiang, Yunjia Song

**Affiliations:** 1 Department of Typhoid, School of Basic Medical Sciences, Heilongjiang University of Chinese Medicine, Harbin 150040, China; sunxutao@hljucm.net; 2 Department of Pharmacology, School of Basic Medical Sciences, Heilongjiang University of Chinese Medicine, Harbin 150040, China; 18741272223@163.com (S.W.); 17737493595@163.com (C.M.); 1577925315@qq.com (Y.Q.); xuzihang9513@163.com (Z.X.); 3 Department of Synopsis of the Golden Chamber, School of Basic Medical Sciences, Heilongjiang University of Chinese Medicine, Harbin 150040, China; xieying@hljucm.edu.cn

**Keywords:** H_2_S donor, ischemia-reperfusion, mechanisms, inflammation, apoptosis

## Abstract

Ischemia–reperfusion (I/R) injury, a prevalent pathological condition in medical practice, presents significant treatment challenges. Hydrogen sulfide (H_2_S), acknowledged as the third gas signaling molecule, profoundly impacts various physiological and pathophysiological processes. Extensive research has demonstrated that H_2_S can mitigate I/R damage across multiple organs and tissues. This review investigates the protective effects of H_2_S in preventing I/R damage in the heart, brain, liver, kidney, intestines, lungs, stomach, spinal cord, testes, eyes, and other tissues. H_2_S provides protection against I/R damage by alleviating inflammation and endoplasmic reticulum stress; inhibiting apoptosis, oxidative stress, and mitochondrial autophagy and dysfunction; and regulating microRNAs. Significant advancements in understanding the mechanisms by which H_2_S reduces I/R damage have led to the development and synthesis of H_2_S-releasing agents such as diallyl trisulfide-loaded mesoporous silica nanoparticles (DATS-MSN), AP39, zofenopril, and ATB-344, offering a new therapeutic avenue for I/R injury.

## 1. Introduction

Ischemia–reperfusion (I/R) is a complex pathological phenomenon characterized by an initial reduction in blood flow to an organ followed by the reintroduction of perfusion and reoxygenation [1,2]. However, the restoration process can cause tissue damage and inflammation. I/R injury can affect multiple organs, including the heart, brain, liver, kidney, intestines, lungs, stomach, spinal cord, testicles, and retina, and is a major contributor to global disability and mortality [3]. The intricate pathophysiology of I/R injury involves inflammatory factors, endoplasmic reticulum stress (ERS), mitochondrial autophagy and dysfunction, apoptosis, and necrosis [4,5,6]. Addressing I/R injury remains a significant challenge in clinical settings, necessitating the development of novel and more effective treatments.

Traditionally considered a toxic gas [7], recent research has identified hydrogen sulfide (H_2_S) as the third gaseous signaling molecule, following carbon monoxide (CO) and nitric oxide (NO), with a broad range of biological effects. H_2_S plays a crucial role in the cardiovascular, nervous, digestive, and reproductive systems [8,9,10]. It provides protection against I/R damage in various organs, including the myocardium, brain, liver, kidney, intestine, lung, stomach, spinal cord, testis, and retina [11,12]. Drugs that regulate H_2_S production levels in the body are used to alleviate I/R damage. To date, various H_2_S donors have been employed to counteract and treat myocardial I/R damage, such as DATS-MSN, AP39, and zofenopril [13,14,15]. Additionally, ATB-344 has been utilized to prevent and treat gastric I/R injury [16]. Consequently, H_2_S contributors may hold promise for treating I/R damage.

This review elucidates the mechanisms by which H_2_S alleviates I/R injury and provides an overview of advancements in using H_2_S donors for treating I/R injury.

## 2. Generation of Endogenous H_2_S

**Figure 1:**
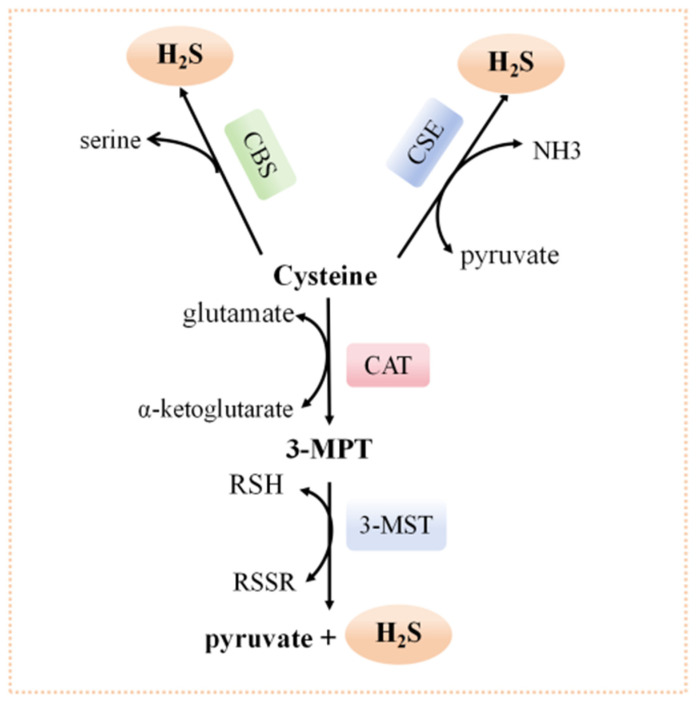
Generation of endogenous H_2_S.

Both endogenous and exogenous H_2_S have demonstrated anti-inflammatory effects and provide protection against I/R injury [7]. Mammalian cells produce H_2_S through both enzymatic and non-enzymatic pathways [17]. Current research primarily focuses on enzymatic pathways [18] (Figure 1). The enzymatic production of H_2_S involves three specific enzymes: cystathionine-β-synthase (CBS), cystathionine-γ-lyase (CSE), and 3-mercaptopyruvate transferase (3-MST) [19]. CBS and CSE, which require pyridoxal-5-phosphate (PLP), generate H_2_S from L-cysteine, whereas 3-MST, which does not require PLP, produces H_2_S from 3-mercaptopyruvate (3-MPT) [8]. These enzymes are distributed in specific tissues within the cytoplasm. CSE is widely present in the heart, liver, kidney, uterus, ileum, placenta, and vascular smooth muscle [20,21]. CBS is highly expressed in the central nervous system, with lower expression levels in the liver, kidney, gastrointestinal tract, uterus, placenta, and pancreatic islets [22]. 3-MST is predominantly found in the cytoplasm and mitochondria, particularly in mitochondria, and is also present in the kidney, heart, liver, lung, central nervous system, and vascular tissues [23].

## 3. H_2_S Donors in I/R injury

H_2_S donors are compounds that release H_2_S under specific conditions [24]. The most common H_2_S donors are sulfide salts such as sodium sulfide (Na_2_S) and sodium hydrosulfide (NaHS), which produce H_2_S rapidly. In contrast, GYY4137, a widely used H_2_S donor in experimental studies, releases H_2_S slowly and continuously. Additionally, diallyl disulfide (DADS) and diallyl trisulfide (DATS), primary allyl sulfur compounds found in garlic, act as natural H_2_S donors [25]. A long-term sustained-release H_2_S system, DATS-MSN, has recently been developed to preserve donor hearts and prevent myocardial I/R injury [13,26]. As a sustained-release H_2_S donor, 5-(4-hydroxyphenyl)-3H-1,2-dithiol-3-thione (ADT) plays a therapeutic role in the blood vessels, kidneys, liver, and intestines [27,28,29,30]. Zofenopril, a thiol-containing angiotensin-converting enzyme (ACE) inhibitor, has been identified as a novel H_2_S donor with significant clinical value in treating cardiovascular diseases [18]. AP39, a mitochondria-targeted H_2_S donor, has been employed to treat myocardial I/R injury [31]. ATB-344, a derivative of indomethacin that releases H_2_S, enhances the gastric mucosa’s defense against gastric I/R damage and releases H_2_S in a dose-dependent manner [16].

## 4. H_2_S and I/R Injury

### 4.1. H_2_S and Myocardial I/R Injury

I/R injury poses a critical challenge in interventional cardiology [32]. Numerous studies have demonstrated the potential of H_2_S and its donors in preventing myocardial I/R damage. Additionally, various drugs can alleviate myocardial injury by upregulating CSE/H_2_S-related pathways.

Luo et al. [33] demonstrated that administering the H_2_S donor NaHS significantly reduced heart injury size, increased left ventricular ejection fraction (LVEF) levels, boosted total antioxidant capacity (T-AOC) and superoxide dismutase (SOD) activity, and lowered reactive oxygen species (ROS) and malondialdehyde (MDA) levels in rats with myocardial I/R injury. Additionally, NaHS therapy elevated H_2_S, CSE, and Bcl-2 (an anti-apoptotic protein) while reducing Bax, caspase-3, caspase-8, and caspase-9 (pro-apoptotic proteins). However, knocking out the *CSE* gene blocked NaHS’s therapeutic effects. In CSE−/− rats with I/R injury, H_2_S and CSE were downregulated, and apoptosis-related proteins were upregulated. These results suggest that I/R injury is linked to the downregulation of the CSE/H_2_S system, where H_2_S upregulation inhibits apoptosis and alleviates oxidative damage in aortic tissues, whereas CSE deficiency exacerbates these effects. In vitro experiments have revealed that H_2_O_2_ treatment reduced H_2_S, CSE, T-AOC, and SOD levels while increasing ROS and MDA levels in human coronary artery endothelial cells (HCAECs). However, treating H_2_O_2_-activated HCAECs with gadolinium chloride (GdCl3), a CaR agonist, notably raised H_2_S, CSE, T-AOC, and SOD levels and reduced ROS and MDA levels. CaR activation increased CSE and H_2_S expression in HCAECs, alleviating oxidative damage. These findings suggest that stimulating the CaR-H_2_S/CSE pathway can protect against myocardial I/R injury by reducing oxidative stress and preventing cardiomyocyte apoptosis. Salloum et al. [34] induced myocardial I/R injury in male CD-1 mice by occluding the left coronary artery for 30 min, followed by 24 h of reperfusion. Beetroot juice (BRJ) administration reduced infarct size by approximately 15% and improved left ventricular remodeling and function. Conversely, administering the CSE inhibitor DL-propargylglycine (PAG) 30 min before ischemia increased infarct size by approximately 48% and negated BRJ’s protective effects. These results suggest that BRJ can prevent myocardial infarction and ventricular dysfunction caused by I/R damage by enhancing CSE-mediated H_2_S production. Using hypoxia followed by reoxygenation, Zheng et al. [35] created an in vitro I/R injury model in H9c2 cells. Hypoxia/reoxygenation (H/R) decreased H_2_S and CSE levels compared to the control group, but trimetazidine treatment counteracted these effects. The *CSE*-knockout group exhibited increased caspase-3 and Bax levels and decreased Bcl-2 levels compared to the trimetazidine-treated group. Additionally, CSE deficiency decreased NADPH oxidase 2 (Nox2), MDA, SOD, and plasma glutathione peroxidase (GSH-Px) activity. These results indicate that trimetazidine protects H9c2 cardiomyocytes against H/R damage by suppressing apoptosis and alleviating oxidative stress via the CSE/H_2_S pathway. Das et al. [36] found that 3′,5′-Cyclic guanosine monophosphate (cGMP)-dependent protein kinase Iα (PKGIα)-overexpression rats had reduced infarct size and maintained left ventricular shortening compared to rats with myocardial I/R injury. PKGIα overexpression increased CSE/H_2_S levels without affecting cardiac CBS and 3-MST expression. However, the protective effects of PKGIα overexpression were eliminated after treatment with the PKG inhibitor KT5823 and the CSE inhibitor PAG. PKGIα overexpression also increased H_2_S levels, decreased lactate dehydrogenase (LDH) levels, and suppressed cardiomyocyte apoptosis compared to control and K390A-treated cardiomyocytes. Treatment with PAG negated PKGIα‘s protective effects and aggravated necrosis, indicating that the benefits of PKGIα genetic treatment for heart I/R damage are linked to increased H_2_S levels. Cinaciguat, a novel soluble guanylate cyclase activator, prevents myocardial I/R damage. Mice treated with 10 μg/kg cinaciguat 30 min before inducing I/R injury had significantly reduced myocardial infarction size and restored left ventricular function [37]. However, treatment with the PKG inhibitor KT5823 and the CSE inhibitor PAG reversed cinaciguat’s therapeutic effects. In vitro, exposing adult ventricular cardiomyocytes to H/R and treating them with cinaciguat increased PKG, cGMP, CSE, and H_2_S levels. These findings indicate that cinaciguat may prevent I/R damage by promoting H_2_S production through the cGMP/PKG pathway. Hu et al. [38] reported that I/R injury groups had lower levels of the *lncRNA Oprm1* compared to control groups. Increased *Oprm1* expression improved heart damage; enhanced heart performance; reduced LDH, ROS, caspase-3, caspase-8, and caspase-9 levels; and increased SOD levels in mice with I/R injury. Furthermore, *Oprm1* elevated the CSE/H_2_S system and reduced miR-30b-5p expression. In vitro, *Oprm1* pretreatment improved cell survival, decreased apoptosis, and increased CSE and H_2_S levels in H9c2 cardiomyocytes exposed to H/R. Inhibition of H/R suppressed phosphatidylinositol 3-kinases (PI3K)/protein kinase B (Akt) and enhanced hypoxia-inducible factor-1 alpha (HIF-1α) and Bcl-2/adenovirus E1B 19kDa interacting protein 3 (Bnip3) levels, inducing apoptosis. Oprm1 pretreatment significantly increased CSE/H_2_S levels, boosted Akt phosphorylation, and reduced HIF-1α and associated signaling molecules. These effects were reversed with *miR-30b-5p* mimics. These findings suggest that *Oprm1* overexpression alleviates myocardial I/R injury by increasing endogenous H_2_S production through the *miR-30b-5p-CSE* axis.

Ustunova et al. [39] found that administering NaSH improved cardiac abnormalities caused by I/R injury and reduced the levels of creatine kinase-MB (CK-MB), LDH, and GPx (indicators of myocardial injury) in a Langendorff perfusion model. The cardioprotective benefits of NaSH were negated by administering the CSE inhibitor PAG, indicating that H_2_S shields the heart muscle from I/R-mediated damage. NaHS reduced the myocardial infarct area by approximately 3%, raised caspase-9 levels, and decreased Bcl-2 expression in rats with local myocardial I/R injury [40]. NaHS reversed cardioprotection and anti-apoptosis effects when treated with the potassium channel blocker 5-hydroxydeoxyate. Additionally, NaHS decreased polymorphonuclear (PMN) and MDA concentrations, suppressed p38 MAPK and c-Jun N-terminal kinase 1/2 (JNK1/2) activation, and prevented NF-κB p65 nuclear translocation compared to the control group. These results indicate that H_2_S plays a cardioprotective role by exerting anti-inflammatory, antioxidant, and anti-apoptotic effects, particularly inhibiting cardiomyocyte apoptosis by activating potassium ion channels and reducing inflammation and oxidative stress by inhibiting the MAPK and NF-κB pathways. Zhang et al. [41] found that cardiac function and arrhythmia scores were enhanced by treatment with 40 μmol/L NaHS, but the protective effects of H_2_S were negated by administering 10 mmol/L glibenclamide, a KATP channel inhibitor. NaHS increased the probability of KATP channel opening, indicating that H_2_S reduces myocardial I/R damage by stimulating the KATP signaling pathway. Li et al. [42] reported that rats with I/R injury exhibited a marked decrease in plasma H_2_S levels. NaSH pretreatment decreased myocardial infarction size, maintained left ventricular function, suppressed apoptosis, and lowered levels of ER/SR stress protein markers Glucose-Regulated Protein 78 (GRP78), CCAAT/enhancer-binding protein homologous protein (CHOP), and ATF6. Tauroursodeoxycholic acid (TUDCA) treatment, which inhibits ER/SR stress, reduced cardiomyocyte apoptosis caused by I/R, and the combined use of NaHS and TUDCA had a synergistic effect in preventing myocardial I/R-induced apoptosis. In vitro tests demonstrated that prior exposure to NaHS effectively inhibited cell death and reduced ER/SR stress protein levels in rat H9c2 cardiomyocytes subjected to H/R stimulation, indicating that H_2_S helps prevent I/R-induced heart damage by reducing ER/SR stress. Xie et al. [43] found that prior treatment with the H_2_S donor ADT lowered mean arterial blood pressure, resulting in decreased myocardial infarct area in rats with I/R damage. ADT’s benefits were nullified by treatment with the AMPK inhibitor compound C (CC). ADT reduced autophagy-related proteins light chain 3-II/I (LC3-II/I), Beclin-1, and P62, and enhanced lysosomal integral membrane protein-2 (LAMP-2) levels. Intravenous CC administration attenuated ADT’s protective effects against I/R-induced impairment of autophagic flux. Administering the autophagy flow blocker chloroquine (CQ) removed ADT’s heart-protective benefits and increased autophagosome buildup in rats with I/R damage. Thus, H_2_S shields the heart from I/R damage by stimulating the AMPK pathway to enhance autophagy.

In ECs dysfunction, the uncoupling of endothelial nitric oxide synthase (eNOS) results in an increase in superoxides (O_2_^−^), a decrease in nitric oxide (NO) production, and a reduction in NO bioavailability [44]. The reaction between superoxide and NO forms the potent oxidants peroxynitrite (ONOO^−^) and hydrogen peroxide (H_2_O_2_) [45]. The diminished availability of NO, along with the elevated levels of H_2_O_2_ and ONOO^−^, can contribute to the development of various vascular diseases, including vascular inflammation, vascular remodeling, and altered vascular tone. Administration of exogenous H_2_S promotes the upregulation of eNOS and NO, leading to increased activity and expression of HIF-1α and VEGF in ischemic mice, thereby facilitating angiogenesis [46]. However, the presence of H_2_S did not influence angiogenesis in *eNOS* knockout mice. Conversely, the absence of CSE in knockout mice hindered NO-induced angiogenesis, suggesting a mutually dependent relationship between the angiogenic effects of H_2_S and NO [47]. Zofenopril, a thiol-containing ACE inhibitor, is a novel H_2_S donor [18]. Donnarumma et al. [15] reported that zofenopril significantly enhanced H_2_S bioavailability in the blood and myocardium and increased NO-2 (a metabolite of NO) levels after 8 h of treatment in mice with I/R damage without significantly altering CSE, CBS, and 3-MST expression. Zofenopril’s role as an H_2_S donor in vivo was the primary reason for improved H_2_S bioavailability, rather than upregulating H_2_S-producing enzymes. Zofenopril and ramipril both decreased infarct size in mice with myocardial I/R injury, but ramipril did not affect plasma and myocardial H_2_S or NO-2 levels. The zofenopril group exhibited a notable rise in the levels of cardiac antioxidant enzymes thioredoxin-1 (TRX-1) and GPX-1 compared to the control group. A clinically relevant I/R injury model was assessed to determine whether zofenopril provided cardiac protection. The results showed that zofenopril pretreatment increased circulating and tissue H_2_S and NO-2 levels, enhanced endothelial NO synthase (eNOS) activation in tissues, decreased infarct size, and inhibited troponin I release in pigs with I/R injury. These findings suggest that zofenopril’s protective effects against I/R injury are associated with increased H_2_S and NO levels. King et al. [48] found that *CSE* knockout resulted in elevated oxidative stress, impaired eNOS function, reduced NO levels, and worsened myocardial I/R damage in mice. Restoration of eNOS/NO bioavailability was achieved with DATS treatment, while Na_2_S provided relief from I/R-induced damage. Thus, H_2_S mitigates I/R injury by stimulating the eNOS/NO system.

Bibli et al. [49] demonstrated that NaSH treatment increased cGMP levels in the heart, elevated phosphorylation (PLN), and reduced infarct size by approximately 35% in rabbits with myocardial I/R injury. The protective effects of NaSH were reversed by treatment with the cGMP-dependent PKG inhibitor DT2. In *PLN*-knockout mice exposed to I/R damage, NaHS did not protect against I/R damage. These findings indicate that H_2_S reduces heart damage caused by I/R by stimulating the cGMP/PKG/PLN pathway. Zhang et al. [50] found that NaSH decreased the infarct area, enhanced cardiac function, and lowered *c-Fos* expression in the myocardium of rats with I/R injuries. Ren et al. [51] showed that H/R exposure led to a notable increase in cell death and upregulation of ERS indicators, including GRP78, CHOP, and eIF2α, in H9c2 rat cardiomyoblasts. However, H_2_S pretreatment reversed H/R-induced apoptosis and ERS. The combination of H_2_S and a miR-133a activator had synergistic protective effects on H/R-stimulated H9c2 cells. Additionally, an I/R injury rat model demonstrated that administering H_2_S and a miR-133a imitator reduced CK and CK-MB levels, elevated LDH levels, and enhanced heart function. These findings indicate that H_2_S shields against I/R-induced ERS and cardiomyocyte death by enhancing *miR-133a* levels. Sodha et al. [52] found that Na_2_S treatment reduced infarct size, enhanced cardiac function, downregulated myocardial injury markers CK-MB and fatty acid binding protein (FABP), and decreased myocardial apoptosis in Yorkshire pigs with myocardial I/R damage. These results suggest that H_2_S effectively suppresses apoptosis induced by I/R damage. Kang et al. [53] discovered that NaHS treatment resulted in a notable decline in apoptotic cells, reduced *miR-1* levels, and increased Bcl-2 *mRNA* and protein expression in rats with I/R injury. Cardiomyocytes isolated from SD rats and stimulated with H/R showed higher cell viability, lower apoptosis rates, reduced LDH and miR-1 expression, and increased Bcl-2 mRNA and protein levels with 30 μmol/L NaHS treatment compared to the H/R group. Further studies indicated that *miR-1* mimic suppressed Bcl-2 levels, suggesting that NaHS inhibits cardiomyocyte death and reduces heart I/R damage by decreasing *miR-1* levels. Hu et al. [54] found that administering NaHS significantly increased silent information regulator sirtuin 1 (Sirt1)/PGC-1α expression, restored Sirt1 nuclear localization, improved heart function, decreased infarct size and myocardial enzyme expression, elevated ATP and SOD levels, and lowered MDA levels in rat hearts with I/R damage. The reversal of NaHS effects due to EX-527 administration indicates that H_2_S may protect the heart against I/R damage by activating the Sirt1/PGC1α pathway. Luan et al. [55] found that 10 μM NaHS notably enhanced heart function while decreasing the infarct size and apoptosis rate in rats with myocardial I/R damage. The Janus kinase 2 (JAK2) inhibitor AG-490 counteracted NaHS’s protective effects. The NaHS group exhibited increased signal transducer and activator of transcription 3 (STAT3) phosphorylation and Bcl-2 expression, as well as decreased Bax expression, compared to the I/R group. These results indicate that H_2_S shields the heart from I/R damage by triggering the JAK2/STAT3 signaling pathway. Treatment with GYY4137 led to a notable improvement in heart function, a reduction in the ischemic area, and a decrease in creatine kinase levels in rats with myocardial I/R damage. GYY4137 increased plasma H_2_S and CSE levels; suppressed serum MDA, myeloperoxidase (MPO), and superoxide anion levels; and inhibited MAPK phosphorylation. It also upregulated Bcl-2 levels while downregulating Bax and caspase-3, leading to the inhibition of myocardial apoptosis [56]. These findings indicate that GYY4137 prevents myocardial I/R damage by reducing oxidative stress and apoptosis. NaSH enhanced cardiac function and reduced damage to myocardial cells and mitochondrial membranes. Compared to the ischemic group, the NaSH treatment group had higher SOD and GSH-Px levels and lower MDA, JNK2, and phosphorylated JNK2 levels. These results indicate that H_2_S might inhibit the JNK pathway to suppress JNK2 phosphorylation and decrease JNK2 protein expression, thereby alleviating oxidative damage caused by myocardial I/R injury [57].

Cardiac mitochondrial dysfunction is a primary manifestation of I/R injury. Cardiac mitochondria are categorized into two types: interfibrous (IFM) and submuscular (SSM). Banu et al. [58] observed that NaSH treatment significantly reduced infarct size, enhanced cardiac function, and decreased LDH and CK expression, markers of myocardial injury, in rats with myocardial I/R injury. Additionally, it increased the activity of all electron transport chain enzymes in both IFM and SSM and alleviated mitochondrial swelling, suggesting that H_2_S enhances cardiac mitochondrial function to mitigate I/R injury. Elrod et al. [59] reported that in Na_2_S-treated mice, myocardial neutrophil infiltration, necrosis, bleeding, and the number of fusiform stromal cells were reduced, while mitochondrial function and membrane integrity were preserved compared to untreated I/R injury mice. Moreover, 10 μM Na_2_S treatment significantly accelerated the recovery of the mitochondrial respiration rate after 30 min of hypoxia in isolated mitochondria from mice, indicating that H_2_S maintains mitochondrial function to protect the heart from I/R damage. Sun et al. [60] found that NaSH treatment decreased ROS and mitochondrial complex IV levels while increasing Mn-SOD levels in H/R-stimulated primary cardiomyocytes, suggesting that H_2_S suppresses mitochondrial complex IV and stimulates superoxide dismutase, thereby reducing ROS levels in heart muscle cells following I/R damage.

Yao et al. [61] observed that H/R accelerated apoptosis; increased the expression of Fas, Fas Ligand (FasL), mitochondrial cytochrome c, and caspase-3; suppressed the phosphorylation of ARC; and enhanced the activity of calcineurin (which dephosphorylates ARC) in rat primary cardiomyocytes. NaHS treatment reduced calcineurin activity and increased casein kinase II (CK2) activity (which phosphorylates ARC), thereby promoting ARC phosphorylation in H/R-stimulated cardiomyocytes. The protective effects of NaHS on cardiomyocytes were reversed by inhibiting DL-alanine glycine with TBB and CSE, specific inhibitors of CK2. These findings suggest that H_2_S inhibits H/R-induced cardiomyocyte apoptosis by enhancing ARC phosphorylation. Yao et al. [62] discovered that administering 30 μmol/kg NaHS to rats with myocardial I/R injury significantly decreased infarct size; reduced myocardial apoptosis; increased levels of phospho-glycogen synthase kinase-3β (p-GSK-3β) Ser9, survivin, and caspase-3; and decreased levels of Bax. Controlled experiments showed that NaHS treatment elevated the phosphorylation levels of GSK-3β (Ser9) and Akt, inhibited Bax translocation, and reduced caspase-3 presence in H/R-stimulated cardiomyocytes. However, GSK-3β inhibition weakened the anti-apoptotic effects of NaHS. These findings suggest that H_2_S inhibits cardiomyocyte apoptosis by increasing GSK-3β (Ser9) phosphorylation, thereby affecting Bax translocation and caspase-3 activation. Moreover, the H/R group required a lower total Ca^2+^ load to open the mitochondrial permeability transition pore (mPTP) compared to the control group, indicating that mPTP opening was facilitated under H/R conditions. NaHS treatment, however, increased the total Ca^2+^ load required for mPTP opening, and this effect was diminished by GSK-3β inhibition. These results suggest that H_2_S prevents Ca^2+^-induced mPTP opening by enhancing GSK-3β (Ser9) phosphorylation, thus attenuating cardiomyocyte apoptosis. Zhou et al. [63] demonstrated that pretreatment with 50 μM NaHS improved left-ventricular-developed pressure (LVDP) and rate pressure product (RPP), enhanced Akt and mechanistic target of rapamycin (mTOR) phosphorylation, and increased Bcl2 expression (a downstream target of Akt) in SD rats with I/R injury. The mTOR inhibitor PP42 counteracted the protective effects of NaHS. NaHS treatment also significantly improved cell survival, reduced early and late apoptosis, decreased LDH expression, and increased Akt phosphorylation at Ser473 and Thr308 in H/R-stimulated H9c2 cells, while PP242 reversed the anti-apoptotic effects of NaHS. These findings indicate that Akt phosphorylation by mTORC2 is a key mediator of H_2_S’s protective effects on myocardial I/R injury.

Xiao et al. [64] discovered that NaHS treatment at doses ranging from 30 to 100 μM/kg decreased the expression of autophagy-related proteins LC3-II, autophagy-related 5 (Atg5), and Beclin1 in rats experiencing I/R injury. Exposure to NaHS at levels between 10 and 100 μM enhanced cell survival and reduced levels of LDH and autophagosome markers in H/R-stimulated myocardial cells isolated from SD rats. The mTOR inhibitor rapamycin counteracted the effects of NaHS, while the autophagy inhibitor 3-methyladenine (3-MA) alleviated cell damage. These findings suggest that H_2_S regulates autophagy by stimulating mTOR, providing protective benefits against myocardial I/R injury. Jiang et al. [65] reported that Atg5, Beclin1, and Atg9 were upregulated in cardiomyocytes isolated from SD rats stimulated with H/R, but downregulated in H/R-stimulated cells treated with 30 μM NaHS. The use of LY294002, an siRNA targeting *Serum glucocorticoid-regulated kinase 1 (SGK1)*, and TWS119, a GSK3β blocker, increased Beclin1 and LC3II/I levels, reduced cell viability, and raised LDH release, diminishing NaHS’s protective effects. H/R decreased t-PI3K and p-PI3K expression, whereas NaHS treatment increased t-PI3K expression. These findings indicate that H_2_S suppresses autophagy through the PI3K/SGK1/GSK3β signaling pathway, ultimately reducing myocardial damage from I/R. Predmore et al. [66] found that administering 200 μg/kg DATS significantly decreased infarct size, maintained heart function, and lowered troponin expression in rats with I/R injury. Additionally, DATS increased eNOS phosphorylation at Ser1177, elevated nitrite and nitrate levels (markers of NO bioavailability), reduced mitochondrial respiration rate in a dose-dependent manner, and enhanced mitochondrial coupling in rats with I/R damage. These findings suggest that DATS stimulates eNOS and enhances NO availability, helping to prevent I/R damage. Sun et al. [13] demonstrated that DATS-MSNs, a novel long-term and slow-releasing H_2_S donor, significantly increased mean arterial pressure (MAP) and Bcl2 levels while reducing CK-MB, troponin I, and Bax levels in rats with myocardial I/R injury. Additionally, MDA levels were lower compared to the NaHS and DATS groups. Myocardial neutrophil infiltration and necrosis were reduced, and MPO expression was downregulated in rats treated with H_2_S donors. The DATS-MSN group showed the most significant reduction in tumour necrosis factor alpha (TNF-α) and IL-1β levels. For H/R-induced cardiomyocyte damage, the DATS-MSN group demonstrated a significant advantage over NaHS and DATS. These findings suggest that DATS-MSNs protect against myocardial I/R injury primarily by inhibiting apoptosis, reducing oxidative stress, and suppressing inflammation. Sun et al. [26] further showed that DATS-MSN suppressed H/R-mediated cardiomyocyte inflammation by inhibiting the toll-like receptor 4 (TLR4)/NACHT, LRR, and PYD domains-containing protein 3 (NLRP3) pathway.

**Figure 2:**
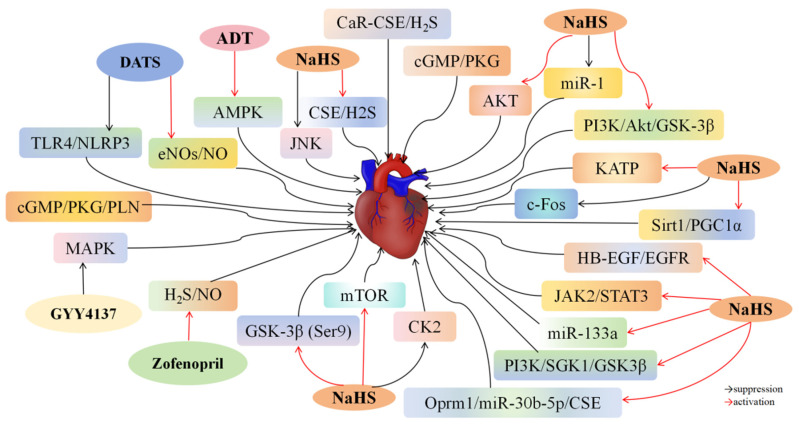
Regulation of H_2_S on myocardial I/R injury.

Studies indicate that older age, obesity, and diabetes are linked to increased severity of myocardial I/R damage [67,68,69]. Ravani et al. [70] found that infarct size in mice with I/R injury decreased with H_2_S donors Na_2_S, GYY4137, and AP39. Obese mice with I/R injury had an infarct size 11% larger than non-obese mice, which was significantly reduced by Na_2_S and AP39 administration, suggesting that H_2_S donors may mitigate myocardial I/R damage regardless of weight. Li et al. [71] showed that ischemic post-conditioning (PC) raised CSE and H_2_S levels while lowering LDH and CK levels (heart damage markers) in young rat hearts, but not in aging rat hearts. NaHS treatment improved heart function, reduced tissue damage, decreased cell death, and lessened oxidative stress in rats of all ages. The cardioprotective effects of NaHS were nullified by LY294002, a PI3K inhibitor, but enhanced by N-acetylcysteine (NAC), a ROS inhibitor. Additionally, external H_2_S administration increased PI3K, Akt, and GSK-3β phosphorylation in rats with I/R damage. Thus, H_2_S may reduce oxidative stress and enhance the PI3K/Akt/GSK-3β pathway, offering potential treatment for heart conditions in older adults. Chen et al. [72] observed that NaHS treatment after 40 min of ischemia significantly alleviated myocardial injury, reduced infarct size, suppressed apoptosis, improved cardiac function, increased autophagy in the aging heart, and restored PC’s cardioprotective effects. The beneficial effects of NaHS were eliminated by 3-Methyladenine (3-MA) treatment. In vitro, D-galactose-induced senescence in H9c2 cells stimulated with H/R was countered by NaHS treatment, which enhanced cell viability and autophagy, attenuated apoptosis, upregulated AMPK phosphorylation, and downregulated mTOR phosphorylation. These findings indicate that H_2_S boosts autophagy via the AMPK/mTOR pathway in aging hearts and cardiomyocytes, reinstating PC’s cardioprotective benefits. Zhang et al. [73] examined the effects of H_2_S on H/R-stimulated H9c2 cells with H2O2-induced senescence. NaHS treatment improved cell morphology, decreased LDH and CK expression, increased cell viability, and suppressed apoptosis. The cardioprotective effects of NaHS were reversed by the epidermal growth factor receptor (EGFR) antagonist AG1478, extracellular signal-regulated kinases 1 and 2 (ERK1/2) inhibitor PD98059, and PI3K inhibitor LY294002. H/R reduced heparin-binding epidermal growth factor-like growth factor (HB-EGF) and EGFR expression in senescent H9c2 cells, which NaHS treatment reversed. AG1478 blocked the increase in HB-EGF and EGFR caused by NaHS, indicating that H_2_S acts through the HB-EGF/EGFR signaling pathway. NaHS treatment in H9c2 cells stimulated with H/R led to increased phosphorylation of ERK1/2, PI3K, Akt, and GSK-3β and enhanced expression of proto-oncogenes *c-myc*, *c-fos*, and *c-jun*. These effects were reversed by PD98059, AG1478, and LY294002 or AG1478 (Figure 2). Overall, the findings suggest that external H_2_S activates the ERK1/2-c-myc, c-fos, c-jun, and PI3K-Akt-GSK-3β pathways in aged cardiomyocytes by enhancing HB-EGF/EGFR signaling (Table 1).

### 4.2. H_2_S and Cerebral I/R Injury

Globally, stroke is the second leading cause of death [118]. Despite various therapies to restore blood circulation to infarcted areas, outcomes are often unsatisfactory due to I/R damage. Ischemia and hypoxia can cause neuronal injury, necrosis, brain tissue damage, and metabolic dysfunction, which are exacerbated by reperfusion. Research indicates that H_2_S can protect the brain from I/R damage.

**Figure 3:**
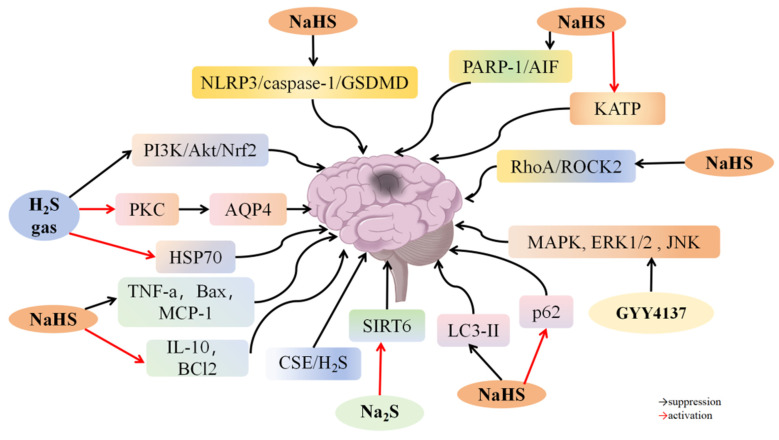
Regulation of H_2_S on cerebral I/R injury.

H_2_S helps reduce brain damage caused by I/R by decreasing neuronal pyroptosis, inflammation, oxidative stress, and cell apoptosis. Gheibi et al. [74] induced focal ischemia through middle cerebral artery occlusion (MCAO) for 60 min, followed by 23 h of reperfusion. Treatment with NaHS at doses of 1 and 5 mg/kg reduced infarct volume by approximately 29% and 51%, respectively, compared to the I/R group. Additionally, it reduced brain swelling and inhibited cell death in rats with I/R damage, suggesting that H_2_S alleviates cerebral I/R damage by attenuating apoptosis (Figure 3). Yang et al. [75] found that administering 50/μM/kg NaHS significantly decreased infarct volume and improved nerve function in rats with cerebral I/R injury. NaHS also suppressed NLRP3 activation and reduced inflammatory factors IL-1β and IL-18, along with downstream effectors gasdermin D (GSDMD) and caspase-1 associated with pyroptosis and apoptosis. These findings suggest that H_2_S may reduce neuronal pyroptosis and inflammation by inhibiting the NLRP3/caspase-1/GSDMD pathway, thereby decreasing cerebral I/R injury. Ji et al. [76] reported that pretreatment with H_2_S reduced escape latency and infarct volume by approximately 25% in mice with MCAO-induced cerebral I/R injury. Compared to the I/R group, the H_2_S treatment group showed lower apoptosis rates, reduced levels of MDA, 8-hydroxy-2′-deoxyguanosine (8-OHdG), IL-6, TNF-α, and caspase-3, and increased 70-kDa heat shock proteins (HSP70) expression in the brain. However, *NF-E2-related factor 2* (*Nrf2)* knockout and the PI3K inhibitor LY294002 decreased HSP70 expression and increased neurological deficits compared to the H_2_S-treated group. These results suggest that H_2_S may reduce cerebral I/R injury by promoting HSP70 expression via the PI3K/Akt/Nrf2 pathway. Han et al. [77] found that GYY4137 administration elevated H_2_S levels in the cerebral cortex of rats with MCAO-induced I/R injury. The GYY4137 and SB203580 groups showed reduced cerebellar infarct volume and increased Garcia scores compared to the I/R group, with the GYY4137 group having a more significant impact. Both groups exhibited lower Bax levels and higher Bcl-2 levels. Additionally, the GYY4137 group had significantly reduced phosphorylation levels of p38 mitogen-activated protein kinase (MAPK), ERK1/2, and JNK proteins compared to the I/R group. These findings suggest that GYY4137 may enhance neural function and decrease infarct size by modulating p38 MAPK, ERK1/2, and JNK signaling pathways to prevent cell death. Zhang et al. [78] found that CSE-KO mice with cerebral I/R injury showed lower motor activity and weaker exploratory and position navigation abilities compared to WT mice. NaHS treatment enhanced exploratory behavior, motor function, and spatial navigation skills in both CSE-KO and WT mice with cerebral I/R damage. Additionally, NaHS reduced neuronal damage; enhanced astrocyte growth; and lowered glial fibrillary acidic protein (GFAP), ras homolog gene family member A (RhoA), and Rho-associated coiled-coil containing protein kinase 2 (ROCK2) levels in the hippocampus. Within a month after cerebral I/R injury, the ROCK inhibitor Fasudil inhibited excessive astrocyte growth and improved neuronal activity. These results indicate that H_2_S reduces cerebral I/R damage by inhibiting the RhoA/ROCK2 signaling pathway, decreasing abnormal astrocyte proliferation, and enhancing neuronal function. Yin et al. [79] found that administering 0.2 or 0.4 mmol/kg NaHS decreased stroke index, neurological symptom score, and infarct size in a dose-dependent manner in rats with cerebral I/R damage. Compared to the I/R group, the NaHS group had reduced levels of MDA and NADPH oxidase p47phox and gp91phox, and increased SOD levels. NaHS also suppressed pro-inflammatory TNF-α and monocyte chemoattractant protein-1 (MCP-1) and increased anti-inflammatory IL-10 expression, lowered Bax levels, and increased Bcl2 levels. These findings suggest that H_2_S decreases oxidative stress and inflammation and suppresses apoptosis in cerebral I/R injury. Wen et al. [80] found that NaHS administration at concentrations ranging from 1 × 10^−5^ to 1 × 10^−7^ mol/kg alleviated diastolic dysfunction in rats with MCAO-induced I/R injury. In *CSE* KO rats, no contractile reaction to MCA was observed, but NaHS at 1 × 10^−6^ mol/kg significantly enhanced the contractile response. NaHS and L-Cys caused MCA dilation, which was reduced by KCa channel blockers ChTx and Apamin. *CSE* KO rats showed higher neurological scores, infarct volumes, cerebral water content, malondialdehyde, and serum lactate dehydrogenase activity, which NaHS supplementation at 1 × 10^−6^ mol/kg reversed. Additionally, *CSE* knockdown in human umbilical vein endothelial cells EAhy926 abolished acetylcholine-induced relaxation. These findings indicate that H_2_S protects against cerebral I/R damage by stimulating potassium channels to mediate vascular endothelial constriction and expansion. Yu et al. [82] discovered that NaHS treatment reduced infarct size and improved neurological severity scores (mNSS) dose-dependently in rats with MCAO-induced cerebral I/R injury. NaHS lowered MDA concentrations, increased SOD and GSH-Px levels, and inhibited apoptosis. In an in vitro model of cerebral I/R injury using HT22 cells with oxygen–glucose deprivation followed by reoxygenation (OGD/R), NaHS treatment decreased ROS levels, suppressed apoptosis, and improved mitochondrial structure and function. NaHS also inhibited poly (ADP-ribose) polymerase-1 (PARP-1) cleavage and apoptosis-inducing factor (AIF) translocation. These findings suggest that H_2_S protects against cerebral I/R injury by reducing oxidative damage and inhibiting cell death caused by PARP-1 cleavage and AIF translocation.

Epidemiological research indicates that alcohol consumption may have a dual impact on the occurrence and outcome of ischemic stroke [83,119,120,121]. McCarter et al. [122] discovered that modest alcohol intake could provide neuroprotection against cerebral I/R injury in rats by reducing post-ischemic inflammation. Their study also revealed that mice with MCAO-induced cerebral I/R injury had increased CSE levels in the cerebral cortex, reduced infarct volume, and enhanced motor function after receiving 0.7 g/kg/d alcohol orally. Alcohol increased H_2_S generation, reduced IL-1 receptor accessory protein and IL-1β levels, and suppressed microglia activation and neutrophil infiltration. CSE inhibitors PAG and β-cyano-l-alanine (BCA) reversed alcohol’s neuroprotective effects against cerebral I/R damage. PAG also counteracted alcohol’s suppressive effects on post-ischemic inflammation [81]. These results suggest that LAC inhibits post-ischemic inflammation by increasing CSE/H_2_S pathway activity, preventing cerebral I/R injury.

H_2_S can reduce cerebral I/R injury by inhibiting autophagy and modulating related signaling pathways. Shui et al. [84] showed that NaHS treatment decreased infarct volume and improved nerve function in KM mice with MCAO-induced cerebral I/R injury. However, the autophagy inducer rapamycin diminished the protective effects of NaHS on infarct volume. Treatment with 3-MA also reduced infarct volume, similarly to NaHS. Additionally, NaHS lowered LC3-II levels and increased the autophagy marker p62. These findings suggest that H_2_S may mitigate cerebral I/R damage by repressing autophagy. Zhu et al. [85] demonstrated that NaHS treatment in KM rats with MCAO-induced cerebral I/R injury decreased infarct volume, reduced LC3-II expression, and increased p62 expression. Inhibition of autophagic flux by bafilomycin A1 (BafA1) nullified NaHS’s protective effects on cerebral infarction. The NaHS group had fewer autophagosomes and early autophagic vacuoles, but more degrading autophagic vacuoles with partially degraded contents compared to the I/R group. These results indicate that H_2_S accelerates autophagy. Increased LC3-II expression, associated with autophagic vacuole degradation, was observed in SH-SY5Y cells after extended OGD/R. However, pretreatment with 200 μM NaHS reduced LC3-II expression, which was fully reversed by co-treatment with BafA1. These findings suggest that increased autophagy is involved in the beneficial effect of H_2_S on cerebral I/R damage. Jiang et al. [86] showed that 5.6 mg/kg NaHS treatment improved nerve function, reduced infarct size, and lowered LDH and caspase-3 expression in rats with MCAO-induced cerebral I/R injury. Compared to the I/R group, the NaHS treatment group exhibited decreased LC3-II/I expression, higher p62 levels, and fewer autophagosomal lysosomes. Additionally, NaHS alleviated cell damage and inhibited autophagy in OGD/R-stimulated PC12 cells. Rapamycin treatment reversed the inhibitory effect of NaHS on LDH and caspase-3 expression, while 3-MA reduced cell damage induced by OGD/R. These findings suggest that H_2_S reduces cerebral I/R damage by inhibiting autophagy.

Expanding blood vessels in the brain provides protection against injury caused by a lack of blood flow and oxygen. Chen et al. [87] discovered that the M-receptor stimulant acetylcholine significantly raised the concentrations of CSE, 3-MST, and H_2_S in vascular endothelial cells (ECs) from primary rat cerebral arteries. However, Atropine, an M receptor antagonist, and PAG, a CSE inhibitor, decreased these expressions. The RhoA agonist U46619 significantly reduced CSE and H_2_S levels in ECs, while treatment with the RhoA inhibitor C3 transferase increased H_2_S levels. NaHS administration relaxed smooth muscle cells in the rat cerebral basal artery by inhibiting ROCK1/2 activity and reducing phosphorylated myosin light chain (MLC) protein expression. Treatment with C3 transferase or the ROCK inhibitor Y27632 reversed these effects. These findings indicate that activation of M receptors in rat brain arteries promotes H_2_S production in ECs. H_2_S donors can induce vasodilation in cerebral blood vessels by inhibiting the RhoA-ROCK pathway, thereby mitigating hypoxia-induced brain damage. Wen et al. [88] reported that CSE−/− mice with MCAO-induced cerebral I/R injury exhibited decreased LDH levels and increased MDA levels compared to sham mice, suggesting that knocking out *CSE* worsened cerebral I/R injury. NaHS upregulated LDH expression and downregulated MDA expression in CSE−/− mice with cerebral I/R damage. Treatment with NaHS induced cerebral basilar artery (BA) relaxation, while the ROCK inhibitor Y27632 significantly counteracted this effect. Prior NaHS treatment suppressed RhoA function and increased ROCK protein levels in BA VSMCs obtained from CSE−/− mice. These findings suggest that the CSE/H_2_S system potentially inhibits cerebral vasodilation by blocking the RhoA/ROCK pathway. Wei et al. [89] found that inhalation of 40 ppm or 80 ppm H_2_S for 3 h at the start of reperfusion significantly enhanced nerve function, decreased infarct size, reduced cerebral edema, and lowered AQP4 expression near the infarct site in rats with MCAO-induced cerebral I/R damage. Inhibition of PKC by Go6983 reversed H_2_S’s beneficial impact on nerves and upregulated aquaporin-4 (AQP4) expression. These findings suggest that H_2_S may reduce cerebral I/R injury by stimulating PKC and reducing AQP4 levels. Hu et al. [90] found that preconditioning with Na_2_S at levels ranging from 25 to 100 mM reduced cell mortality and lactate dehydrogenase levels in OGD/R-stimulated cerebral ECs in a dose-dependent manner. However, treatment with PAG aggravated OGD/R-stimulated cell death. Na_2_S at 50 mM inhibited intracellular ROS generation and enhanced SOD and catalase (CAT) function in ECs stimulated by OGD/R. It also increased SIRT6, CSE, and H_2_S levels in OGD/R-stimulated ECs, while treatment with PAG reversed this effect. Moreover, *SIRT6* knockdown counteracted Na_2_S’s effects on LDH, SOD, CAT, and cell death. These findings suggest that Na_2_S protects brain ECs from I/R damage by enhancing SIRT6 levels.

### 4.3. H_2_S and Hepatic I/R Injury

An I/R injury to the liver typically occurs during surgery, leading to inflammation, oxidative stress, and apoptosis [123,124]. Factors such as aging and fatty liver exacerbate liver failure in cases of I/R injury [125]. Therefore, developing effective treatment strategies for hepatic I/R injury is essential. Several studies have demonstrated that H_2_S can protect the liver from I/R injury.

**Figure 4:**
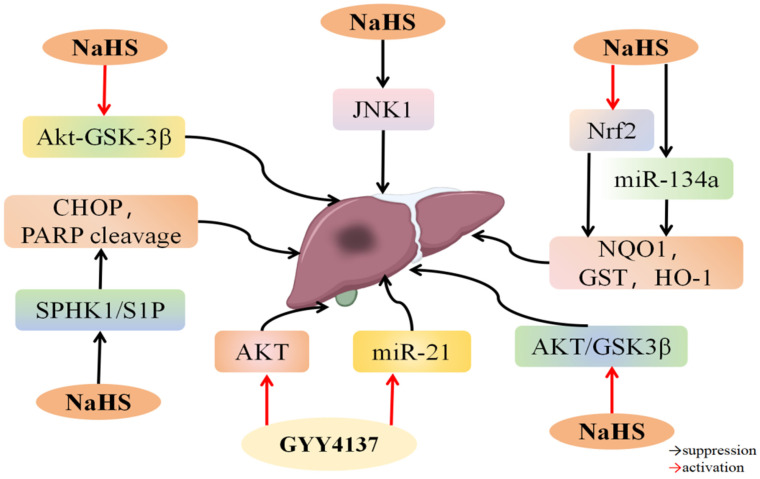
Regulation of H_2_S on hepatic I/R injury.

*miRNAs*, which are non-coding *RNAs* ranging from 20 to 25 nucleotides in length, regulate various biological functions, and their irregular levels are linked to numerous health issues, including liver diseases [126]. Research has demonstrated that *miR-34a* is involved in safeguarding liver function [127]. Increased *miR-34a* levels are associated with age-related changes in hepatic antioxidant activity. *Nrf-2*, a gene targeted by *miR-34a*, controls genes that help combat oxidative stress, including *glutathione S-transferase (GST)*, *SOD*, *heme-oxygenase-1(HO-1)*, and *NAD(P)H quinone oxidoreductase 1 (NQO1)* [128,129,130]. Huang et al. [91] induced hepatic I/R injury in rats of varying ages and found that NaHS treatment notably reduced aspartate aminotransferase (AST) and alanine aminotransferase (ALT) levels, indicators of liver function, in young rats with hepatic I/R injury, while only marginally affecting these markers in elderly rats. The NaHS treatment group exhibited higher levels of *miR-34a* and lower levels of Nrf-2, along with reduced expression of its downstream targets NQO1, GST, and HO-1, compared to the I/R group. Increased *miR-34a* levels decreased Nrf-2 and its target genes *NQO1*, *GST*, and *HO-1*, counteracting the protective benefits of H_2_S in preventing liver damage from I/R. However, blocking *miR-34a* led to a rise in Nrf-2, NQO1, GST, and HO-1 levels, amplifying the beneficial impact of H_2_S on elderly rats experiencing liver damage from I/R (Figure 4). These findings indicate that H_2_S attenuates liver I/R damage in rats of varying ages by increasing Nrf-2 levels and decreasing *miR-34a* levels. Specifically, the beneficial impact of H_2_S on hepatic I/R injury is influenced by the miR-34a-driven Nrf-2 pathway.

Several studies have shown that blocking the PI3K/Akt, MAPK, and other apoptosis-regulating pathways can effectively improve the inflammatory response triggered by hepatic I/R damage [131]. The balance between Bax and Bcl-2 proteins determines cell survival or apoptosis following stimulation or damage. Unlike Bax, Bcl-2 suppresses apoptosis by inhibiting its release and oligomerization. The correlation between Bax and Bcl-2 protein levels is crucial for triggering cell death through apoptosis following I/R injury [92,131]. JNK1 is a highly efficient pathway for cell survival and is crucial for repairing hepatocytes, making its regulation a promising strategy for alleviating liver injury [132]. Tsung’s study found that hepatic I/R damage activates the JNK signaling pathway, but blocking this pathway reduces liver cell apoptosis caused by I/R injury [133]. According to Chen et al. [134], the I/R group showed increased p-JNK1 levels, but prior treatment with NaHS led to dephosphorylation of JNK1 and ERK1. Treatment with the JNK inhibitor SP600125 suppressed autophagy by inhibiting the JNK pathway, thereby enhancing the hepatoprotective effects of NaHS. Moreover, phosphorylation of Bcl-2 by JNK1 significantly decreased the binding between Bcl-2 and Beclin-1, leading to the rapid separation of Bcl-2 from Beclin-1. This separation triggered protective autophagy and alleviated liver I/R damage. These findings indicate that H_2_S helps to reduce liver I/R damage by blocking the JNK1 signaling pathway and controlling cell death.

Evidence suggests that H_2_S alleviates I/R-induced liver injury by activating the Akt pathway [135,136,137,138]. *MiR-21* activates the Akt pathway to regulate PTEN expression, resulting in anti-inflammatory and anti-apoptotic effects [138,139,140,141]. Meng et al. [93] revealed that elevated H_2_S concentrations led to the upregulation of *miR-21* and controlled Akt pathway activation by downregulating phosphatase and tensin homolog (PTEN) expression. Activated Akt inhibits GSK3β by increasing its phosphorylation, which in turn reduces caspase-9 activation and inhibits caspase-3. Additionally, blocking GSK3β prevents the release of inflammatory cytokines by triggering transcription factors. Thus, H_2_S can upregulate *miR-21*, activate the Akt pathway, and exert anti-apoptotic effects to mitigate hepatic I/R damage [142]. Sameri et al. [94] found that NaHS treatment reduced inflammatory cytokine levels, decreased apoptosis, and lowered hepatic total oxidants and aminotransferase levels, enhancing liver function in mice with I/R injury. Furthermore, pretreatment of H/R-stimulated mesenchymal stem cells (MSCs) derived from human umbilical cords with NaHS enhanced the therapeutic effects of MSC-derived exosomes. These findings indicate that H_2_S may reduce liver I/R damage and improve the healing benefits of exosomes.

ERS is a unique cytopathological state marked by disrupted cell homeostasis and ER dysfunction. Activation of PERK, inositol requiring enzyme 1 (IRE1), and ATF6 by ERS triggers the unfolded protein response (UPR) to restore ER balance [143]. Emadali et al. [144] found that the UPR pathway was activated in hepatic I/R injury, indicating the significant role of ERS. While UPR prevents excessive cell damage, persistent ERS induces severe inflammation and apoptosis. CHOP, a crucial transcription factor, can trigger cell death under prolonged ERS. Chen et al. [95] demonstrated that NaHS administration reduced hepatic I/R damage by lowering the levels of ERS-related proteins and *mRNA*, including ATF6, PERK, GRP78, tumor necrosis factor receptor-associated factor 2 (TRAF2), and CHOP. Conversely, blocking H_2_S production exacerbated liver damage from I/R. These findings indicate that H_2_S protects the liver from I/R damage by alleviating ERS and reducing cell mortality. Sphingosine-1-phosphate (S1P), a bioactive lipid, influences processes like angiogenesis, cell movement, immune cell trafficking, and inflammation. Activated by sphingosine kinase 1 (SPHK1), S1P is vital in chronic liver damage development. Targeting the SPHK1/S1P pathway offers a potential way to treat hepatic I/R injury [145]. Qi et al. [146] found that the SPHK1/S1P pathway suppressed CHOP expression, while increased IRE1α levels diminished SPHK1’s protective effects against palmitic acid-induced apoptosis. Chen et al. [96] observed that NaHS treatment reduced inflammatory factors in hepatic I/R damage, while the ER activator PR-619 intensified inflammation. NaHS pretreatment lowered CHOP, SPHK1, and S1P expression compared to the I/R group. However, PR-619 reversed NaHS’s inhibitory effects on these proteins. These findings suggest that H_2_S protects against hepatic I/R injury by inhibiting the SPHK1/S1P pathway and alleviating ERS.

Hepatic I/R injury is associated with the activation of mPTP, leading to an influx of solutes and water, disintegration of the outer mitochondrial membrane, and necrotic cell death. Prior research has demonstrated that activating the PI3K/Akt pathway can block mPTP opening, thereby reducing hepatic I/R damage [147,148,149]. Zhang et al. [97] found that administering NaHS to rats protected them from I/R-induced liver damage by lowering blood ALT and AST levels and preserving normal liver cell morphology. Additionally, NaHS significantly elevated Bcl-2 and p-Akt levels and promoted the phosphorylation of Akt and GSK-3β at Ser9. Phosphorylation of GSK-3β at Ser9 facilitates its binding to mPTP regulators, preventing mPTP opening during I/R injury. In summary, it activates the Akt-GSK-3β signaling pathway, increasing Bcl-2 protein levels, preventing mPTP opening, and reducing the activation of the cytochrome c-caspase-3/9 apoptosis pathway, ultimately protecting liver cells from I/R damage.

### 4.4. H_2_S and Renal I/R Injury

**Figure 5:**
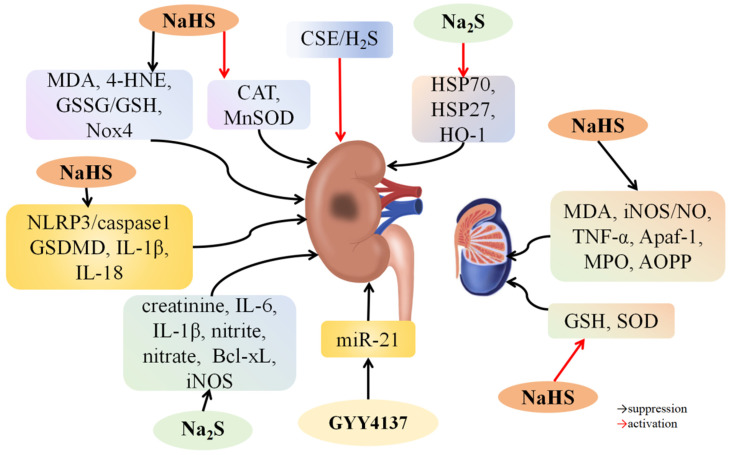
Regulation of H_2_S on renal and testicular I/R injury.

I/R can cause acute kidney injury, induce adverse reactions after transplantation, and increase morbidity and mortality [150]. H_2_S has shown potential in protecting kidneys from I/R damage. Han et al. [99] found that 30 min of bilateral renal ischemia in male C57BL/6 mice resulted in flattened renal tubules, ruptured tubular epithelial cells, increased interstitial cells, and decreased levels of CSE, CBS, and H_2_S. Administering NaHS restored kidney function and tubular structure, effects that were reversed by PAG, a CSE inhibitor. NaHS also accelerated renal tubular cell proliferation and inhibited interstitial cell proliferation. The NaHS group exhibited lower levels of MDA, 4-hydroxy-2-nonenal (4-HNE), lipid peroxidation, glutathione disulfide (GSSG)/GSH, and NADPH oxidase 4 (Nox4), and higher levels of CAT and MnSOD were found compared to the I/R group (Figure 5). These findings suggest that H_2_S mitigates kidney injury by reducing oxidative stress caused by I/R injury. IFC-305, an adenosine derivative, regulates H_2_S expression by inhibiting methylation at the CSE promoter region. Jiang et al. [100] reported that IFC-305 administration alleviated kidney damage and reduced creatinine levels in rats with renal I/R injury. In these rats, renal tissue and peripheral blood homocysteine (Hcy) levels were elevated, and CSE expression was lowered. Treatment with IFC-305 decreased Hcy levels and increased CSE expression, reduced DNA methylation levels of CSE promoters, and lowered MDA and superoxide levels in kidney tissues. In vitro, Hcy treatment increased CSE activity and DNA methylation levels of CSE promoters in HK-2 cells, while IFC-305 treatment had the opposite effect, lowering MDA and superoxide levels in IFC-305-treated HK-2 cells compared to Hcy-treated cells. These findings indicate that IFC-305 alleviates renal I/R damage by blocking CSE promoter methylation to increase H_2_S levels. Norswertianolin (NW), a novel small-molecule agonist of CSE, was shown by Niu et al. [101] to reduce renal I/R damage by elevating H_2_S levels and lowering blood urea nitrogen (BUN) and serum creatinine (Cr) levels, reliable markers for renal function. These findings suggest that NW protects against renal I/R damage by enhancing the CSE/H_2_S pathway.

H_2_S mitigates kidney I/R damage by reducing inflammation, oxidative and nitrosative stress, pyroptosis, and apoptosis while modulating associated signaling pathways. Pushpakumar et al. [98] observed that old mice with renal I/R exhibited decreased plasma H_2_S levels and increased *miR-21* upregulation compared to young mice. GYY4137 treatment restored plasma H_2_S levels and decreased *miR-21* expression in both age groups with I/R injury. Elderly kidneys showed higher macrophage polarization towards the M1 inflammatory phenotype, increased cytokine expression, endothelium–interstitial transformation, and fibrosis compared to young I/R kidneys. Administering 0.25 mg/kg of GYY4137 or a 20 mg/kg *miR-21* antagonist improved kidney health, enhancing renal vascular density, blood circulation, and function in elderly mice. These findings indicate that H_2_S reduces macrophage-induced kidney injury during I/R by blocking *miR-21*. Simon et al. [102] discovered that Na_2_S administration mitigated glomerular tissue injury and decreased creatinine, IL-6, IL-1β, nitrite, and nitrate levels in pigs with renal I/R injury. Additionally, it reduced Bcl-xL and iNOS expression and alleviated oxidative DNA damage. These results suggest that H_2_S alleviates tissue damage and organ dysfunction caused by inflammation, oxidative stress, and nitrosative stress during renal I/R damage. Du et al. [103] found that renal I/R in rats caused tubular enlargement, blockage, bone marrow bleeding, cell swelling, and tubular cell death. It increased serum creatinine and BUN levels, upregulated MDA, promoted lipid peroxidation, and downregulated SOD. It also triggered an inflammatory response and promoted kidney cell death by upregulating NF-қB, IL-2, and TLR4. NaHS administration reversed these effects and upregulated HSP70, HO-1, and HSP27 in rats with I/R damage. Subsequent treatment with the HSP inhibitor quercetin reversed NaHS’s protective effects on the kidneys. These findings indicate that H_2_S benefits lipid peroxidation, inflammation, and apoptosis resulting from renal I/R injury by enhancing heat shock protein 70 (HSP70), HO-1, and heat shock protein 27 (HSP27) levels. Ni et al. [104] observed that renal I/R injury in mice resulted in elevated BUN and Cr levels, severe renal tubular dilation, cell swelling, epithelial cell detachment, necrosis, and inflammatory cell infiltration compared to control rats. Additionally, H_2_S, CSE, and CBS concentrations in kidney tissues of the I/R group were lower than in the control group. NaHS treatment notably alleviated renal I/R injury, while PAG treatment worsened it. NaHS decreased pyrogenic cells and inhibited caspase-1, GSDMD, IL-1β, and IL-18 levels. In HK2 cells, H/R stimulation increased caspase-1, GSDMD, IL-1β, and IL-18 levels and LDH activity, which NaHS treatment partially reversed. Additionally, H/R stimulation upregulated NLRP3 and ASC proteins, which NaHS treatment downregulated. Blocking the NLRP3 inflammasome with MCC950 counteracted NaHS’s beneficial impact on HK2 cells. These findings indicate that H_2_S reduces kidney I/R injury by blocking the NLRP3/caspase-1 pathway to prevent cell death.

### 4.5. H_2_S and Testicular I/R Injury

Testicular torsion is a sudden urogenital issue caused by the twisting of the testicle around the spermatic cord, potentially leading to lasting damage. Temporary blood flow loss can cause this twisting, which may result in infertility even after successful treatment. The testis is highly sensitive to increased oxygen free radicals, making I/R injury a major factor affecting normal testicular function [151]. Yuksel et al. [152] found that NaHS treatment significantly suppressed MPO activity and advanced oxidation protein product (AOPP) levels while enhancing GSH levels and promoting spermatogenic cell activity in rats with testicular torsion-induced I/R damage (Figure 5). Bozkurt et al. [153] discovered that NaHS treatment inhibited MDA, iNOS/NO, and TNF-α levels while increasing GSH and SOD levels in rats with testicular torsion-stimulated I/R damage. Additionally, it inhibited germ cell apoptosis by decreasing the expression of Apaf-1 in rats with testicular I/R damage. These findings suggest that H_2_S may reduce I/R injury caused by testicular torsion through its anti-inflammatory, antioxidant, and anti-apoptotic properties.

### 4.6. H_2_S and Gastric I/R Injury

**Figure 6:**
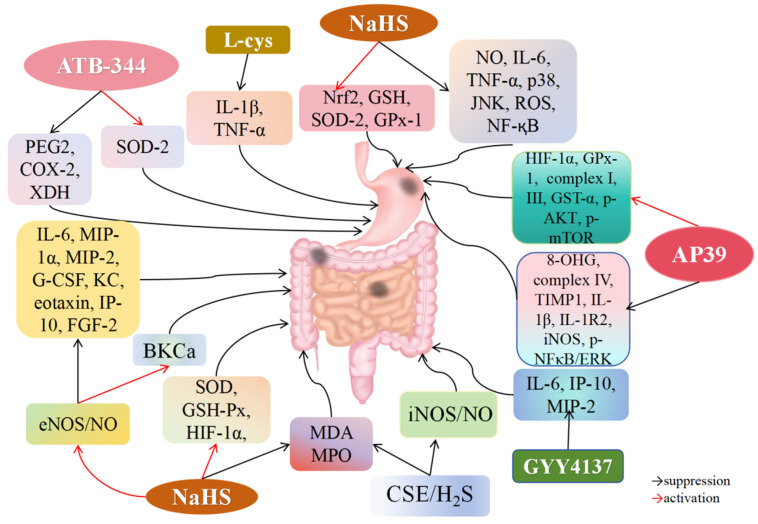
Regulation of H_2_S on gastric and intestinal I/R injury.

Gastric I/R damage can arise from conditions such as hemorrhagic shock, bleeding ulcers, vascular tears, surgical procedures, and gastrointestinal ischemic disorders, leading to high incidence and mortality rates. However, effective clinical treatments for gastric I/R damage are currently lacking [154,155]. The pathogenesis of gastric I/R injury is closely associated with oxidative stress, inflammation, apoptosis, and mucosal injury [156]. Excessive ROS production is considered a key pathogenic factor in gastric mucosal I/R injury [157]. H_2_S is recognized for its ability to protect against gastric I/R injury by reducing oxidative stress and exhibiting anti-inflammatory properties [158,159,160,161]. Mard et al. [105] clamped the abdominal artery in Wistar rats for 30 min, followed by 3 h of reperfusion. NaHS or L-cysteine treatment reduced the size of gastric mucosal injuries and lowered IL-1β and TNF-α levels caused by I/R, while PAG administration counteracted these protective effects and increased inflammatory markers (Figure 6). These results suggest that H_2_S alleviates gastric I/R injury by inhibiting pro-inflammatory mediators. SOD-2, a mitochondrial enzyme, converts superoxide into hydrogen peroxide, reducing peroxide radicals [162,163,164,165]. Lack of SOD-2 in mitochondria elevates ROS generation, disrupting the mitochondrial metabolism and cellular redox balance [166]. Gowacka et al. [16] found that ATB-344, an indomethacin derivative and novel H_2_S-releasing drug, significantly reduced gastric lesion size in rats with gastric I/R injury. The ATB-344 treatment group showed reduced 8-amino-3,6-dioxaoctanoic acid (PEG2) and cyclooxygenase-2 (COX-2) levels in the gastric mucosa compared to the I/R group and increased SOD-2 levels while decreasing xanthine dehydrogenase (XDH) and inflammation markers. These findings suggest that ATB-344 protects against gastric I/R damage through its anti-inflammatory and antioxidant properties. Guo et al. [106] investigated H_2_S’s protective properties against I/R damage in gastric epithelial cells. NaHS treatment significantly enhanced GSH levels and suppressed MDA, NO, IL-6, TNF-α, and ROS in I/R-stimulated gastric epithelial cells, preventing oxidative stress and inflammation. H_2_S induced Keap1 dissociation from Nrf2 and activated Nrf2 through Keap1 S-sulfhydration. Additionally, H_2_S inhibited p38, JNK-dependent apoptosis, and NF-κB-dependent inflammatory pathways. Overall, exogenous H_2_S prevents gastric I/R injury by reducing apoptosis, enhancing ROS elimination, and blocking inflammatory cytokine secretion. Magierowski et al. [107] found that L-cysteine, NaHS, and GYY4137 treatments reduced the gastric damage area in rats with I/R. NaHS treatment increased SOD-2 and GPx-1 levels in rats with gastric I/R damage. These results suggest that H_2_S improves gastric I/R damage through antioxidant properties. Magierowska et al. [108] found that H_2_S-prodrug AP39 pretreatment significantly reduced the gastric lesion area and increased gastric blood flow in rats with I/R damage. AP39 increased levels of HIF-1α, GPx-1, ATP production, mitochondria complex I, complex III, GST-α, p-AKT, and p-mTOR while reducing levels of 8-OHG, complex IV, tissue inhibitor of matrix metalloprotease 1 (TIMP1), IL-1β, type 2 IL-1 receptor-2 (IL-1R2), iNOS, p-NF-κB, and p-ERK in the gastric mucosa. The protective effect of AP39 on gastric I/R injury was reversed by the mTOR inhibitor rapamycin. These results indicate that AP39 activates mTOR and suppresses NF-κB signaling to relieve gastric mucosal inflammation and oxidative damage caused by I/R.

### 4.7. H_2_S and Intestinal I/R Injury

Intestinal I/R injury involves a complex pathological process including calcium overload, oxidative stress, ERS, mitochondrial dysfunction, apoptosis, protein kinase activation, and inflammation. Neutrophil activation, mucosal epithelial cell damage, increased ROS levels, and the release of cytotoxic factors initiate an inflammatory cascade. ROS-induced inflammation enhances lipid peroxidation, worsens oxidative stress-induced tissue damage, and increases MDA production [167]. Intestinal I/R injury results from oxidative stress and an excess of free radicals. H_2_S can enhance long-term potentials by activating MDA receptors, regulate the redox state, maintain excitatory/inhibitory neurotransmission balance, and alleviate oxidative damage by clearing free radicals and active substances [168]. Biosynthesis of NO is required for H_2_S-induced angiogenesis, and eNOS inhibition can abolish this response. Intestinal I/R injury, common in patients with coagulopathy, mechanical obstruction, and severe trauma, can lead to multiple organ dysfunction syndrome. Liu et al. [109] found that NaHS lowered MDA levels and raised SOD and GSH-Px levels, reducing the severity of intestinal I/R injury in rats (Figure 6). These findings suggest that H_2_S serves as a potent antioxidant modulator, protecting against intestinal I/R injury by increasing antioxidant enzyme levels. Furthermore, H_2_S-induced vasodilation requires endogenous NO. Neutrophil recruitment during intestinal ischemia-induced inflammation relies on the chemokines macrophage inflammatory protein-2 (MIP-2) and interferon-γ-induced protein 10 (IP-10). IL-6 promotes intestinal hyperplasia and villi growth, while IL-10 is significantly downregulated in intestinal ischemia [169,170]. Jensen et al. [110] demonstrated that NaHS treatment enhanced mesenteric blood flow and reduced mucosal damage in mice with intestinal I/R injury, but not in eNOS-KO mice. NaHS reduced inflammatory cytokines such as IL-6, MIP-1α, MIP-2, granulocyte colony-stimulating factor (G-CSF), CXCL1 (KC), eotaxin, and IP-10, as well as growth factors like fibroblast growth factor 2 (FGF-2), with these effects reversed in eNOS-KO mice. These findings indicate that H_2_S mitigates intestinal I/R damage and inflammation through the regulation of eNOS/NO pathways in endothelial cells. Jensen et al. [171] further found that pre-ischemic administration of the H_2_S donor GYY4137 improved mesenteric perfusion; alleviated intestinal mucosal damage; and suppressed IL-6, IP-10, and MIP-2 levels. Wang et al. [111] discovered that resveratrol (RES) and RES/PEGyl-polyphenylalanine (RES/PEG-PPhe) reduced intestinal I/R damage in diabetic rats. Diabetic rats with intestinal I/R damage showed upregulated iNOS/NO and MDA levels, reduced SOD activity and GSH levels, and lowered CSE expression. PAG aggravated I/R injury, while iNOS inhibitors offered protection, indicating that endogenous H_2_S protects against intestinal I/R injury in diabetic rats. Prior administration of RES and RES/PEG-PPhe enhanced CSE/H_2_S levels and reduced iNOS/NO levels. These findings indicate that RES/PEG-PPhe reduces intestinal I/R damage in diabetic rats by modulating the CSE/H_2_S and iNOS/NO pathways.

It has been revealed that H_2_S protects intestinal mucosa integrity by inhibiting inflammatory responses and apoptosis, facilitating repair, restoring homeostasis, promoting vasodilation, and improving mesenteric blood flow [172,173]. Additionally, H_2_S enhances the vasodilator effect of NO, which regulates H_2_S production to increase the activity of endothelial BKCa channels and voltage-gated and calcium-activated potassium channels involved in NO synthesis. Zuidema et al. [112] demonstrated that NaHS suppresses inflammation through BKCa channel activation. NaHS preconditioning 24 h before intestinal I/R damage induced capillary BKCa α subunit expression, preventing leukocyte rolling and adhesion during reperfusion. BKCa channel stimulation may increase endothelial cell growth, boost calcium entry via non-voltage-gated calcium channels, and stimulate NO production via calcium-sensitive eNOS. These findings indicate that H_2_S promotes anti-adhesion characteristics via BKCa channels, providing anti-inflammatory benefits to protect the intestines from I/R damage.

Cardiac arrest and cardiopulmonary resuscitation (CPR) can cause intestinal I/R damage. Pan et al. [113] found that CPR induced intestinal mucosal injury in rats, while NaHS treatment alleviated this damage. NaHS reduced MDA and MPO concentrations, elevated SOD and GSH levels, and inhibited apoptosis of intestinal cells following CPR. Further analysis revealed that NaHS increased HIF-1α levels in the intestinal region post-CPR. These findings indicate that H_2_S protects the intestinal mucosa from CPR-induced damage by increasing HIF-1α expression, decreasing oxidative stress, reducing inflammation, and preventing apoptosis.

### 4.8. H_2_S and Lung I/R Injury

Lung I/R damage is a significant issue following lung transplants and the use of heart–lung machines [174,175,176]. Multiple studies have shown that H_2_S can reduce lung damage induced by liver I/R. Fu et al. [114] found that CSE and H_2_S were downregulated in lung tissues collected from rats with I/R damage. Exogenous H_2_S treatment improved lung I/R injury, while the CSE inhibitor DL-propargylglycine (PPG) worsened it. Additionally, H_2_S administration lowered MDA levels and increased SOD and CAT levels in lung tissues, mitigating oxidative stress. These findings suggest that the CSE/H_2_S pathway is involved in lung I/R injury, and H_2_S treatment can protect the lungs from I/R-induced damage. Lung injury caused by lower limb I/R (LIR) can lead to lung dysfunction, characterized by systemic inflammation, pulmonary interstitial edema, and oxidative response. Qi et al. [115] induced lung damage in SD rats through LIR, resulting in alveolar congestion, structural destruction, wall thickening, and inflammatory cell infiltration. NaHS treatment alleviated lung injury, while PPG treatment exacerbated it. Furthermore, NaHS treatment elevated AQP1/AQP5 levels and reduced TLR4 and Myd88 levels. These findings indicate that H_2_S reduces LIR-induced lung injury by regulating the TLR4-Myd88 pathway and AQP1/AQP5 expression to lessen inflammation.

### 4.9. H_2_S and Spinal Cord I/R Injury

Spinal cord I/R (SCI/R) damage, a serious condition causing the loss of sensory and motor functions, primarily affects individuals with spinal injuries, spinal deterioration, or intraspinal growths, and is a common issue following thoracic and abdominal aorta surgery [177,178,179]. The pathogenesis of SCI/R injury is complex, involving alterations in cell membrane permeability, disruptions in water and electrolyte balance, and lipid oxidation [180,181]. Effective treatments for SCI/R injury are necessary. Studies have shown that *miRNAs* play a role in I/R injury. *miR-30c* can protect the brain by targeting brain-derived neurotrophic factors and axon orientation [181]. Additionally, certain *miRNAs* regulate autophagy through modulation of autophagy-associated genes [182]. *Beclin-1-induced* autophagy significantly influences cell survival and death, while *light chain 3 (LC3)* enhances autophagy by converting *LC3-I* into *LC3-II* [183]. Li et al. [116] demonstrated that NaHS treatment enhanced motor function, decreased infarct area, downregulated *miR-30c*, and activated autophagy by increasing Beclin-1 and LC3II in rats with SCI/R injury. Moreover, prior treatment with pre-miR-30c or autophagy inhibitor 3-MA reversed the beneficial effects of H_2_S on SCI/R injury. An in vitro SCI/R injury model was created by inducing OGD in the hippocampal neuron line SH-SY5Y. Consistently, NaHS reduced *miR-30c* levels and enhanced Beclin-1 and LC3II expression in OGD-induced cells. However, pretreatment with miR-30c decreased *Beclin-1 mRNA* expression. These findings indicate that H_2_S protects against SCI/R injury and enhances motor function by suppressing autophagy via a *miR-30c-related* mechanism.

*miR-485-5p* promotes *mRNA* degradation and blocks translation by targeting the 3′-UTR of genes, a process affecting SCIR injury [184]. TNF-α, a key mediator of neuroinflammation, interacts with receptors on the cell surface and contributes significantly to nerve cell apoptosis [185,186]. Numerous studies have shown that TNF-α is stimulated by SCI/R injury and serves as a promising therapeutic target [187,188]. TRADD, a downstream factor of TNF-α, mediates TNF-α-induced cell survival pathways in tumor and nerve cells [189,190]. This protein is highly regulated and efficient in the human nervous system, primarily mediating the TGF-α signaling pathway [191,192]. TRADD interacts with TGF-α, functioning as a signal response factor in cell proliferation and apoptosis. Chen et al. [193] found that patients with SCI/R damage exhibited higher TGF-α and lower *miR-485-5p* levels than healthy controls. Additional in vitro SCI/R models using TNF-α-stimulated human neural cell lines AGE1.HN and SY-SH-5Y showed that GYY4137 had protective effects on TNF-α-stimulated nerve cells, involving the *miR-485-5p-mediated* TRADD signaling pathway in nerve injury repair. GYY4137 treatment inhibited TRADD expression, an effect attenuated by blocking *miR-485-5p*. These findings indicate that GYY4137 suppresses TRADD by enhancing *miR-485-5p*, providing superior protection against SCI/R damage.

### 4.10. H_2_S and Retinal I/R Injury

Retinal damage from I/R can lead to neuron death through apoptosis and necrosis activation [194,195]. Retinal ganglion cells (RGCs) are particularly vulnerable to ischemia compared to other retinal neurons [196,197]. Previous studies have shown that retinal tissue has good tolerance to short-term ischemia [194,198]. Biermann et al. [117] found that administering H_2_S before retinal ischemia attenuated RGC necrosis and apoptosis and inhibited caspase-3 activity, thus playing an anti-apoptotic role. Prior exposure to H_2_S also reduced the activation of glial fibrillary acidic protein (GFAP) and increased HSP-90α protein levels. Additionally, it lowered the DNA-binding capacity of NF-κB and reduced JNK phosphorylation levels in the ischemic group compared to the I/R group. These findings indicate that prior exposure to H_2_S can prevent retinal I/R damage by blocking cell death.

## 5. Conclusions and Perspective

The review highlights recent advancements in understanding how H_2_S mitigates I/R injury across various organs, including the heart, liver, kidney, brain, intestine, stomach, lung, testis, and retina. H_2_S reduces I/R damage by modulating inflammatory factors, ERS, mitochondrial autophagy, dysfunction, apoptosis, necrosis, and associated signaling pathways. Specifically, H_2_S increases SIRT6 expression by activating potassium ion channels and various signaling pathways, such as eNOS/NO, cGMP/PKG/PLN, Sirt1/PGC1α, JAK2/STAT3, TLR4/NLRP3, HB-EGF/EGFR, and PI3K/Akt/Nrf2. H_2_S decreases inflammation resulting from I/R injury by inhibiting pathways like MAPK, NF-κB, JNK, NLRP3/caspase-1/GSDMD, and RhoA/ROCK2, as well as by reducing levels of *c-Fos*, *miR-1*, and *miR-21*. It also reduces oxidative stress by enhancing hsp70, HO-1, hsp27, HIF-1α, Nrf2, and AQP1/AQP5, along with TLR4-Myd88-NF-ĸB pathway function. H_2_S improves cardiac mitochondrial function by activating TLR4/NLRP3 and AKT-GSK-3β, thus preventing I/R damage. It suppresses ER/SR stress and apoptosis by upregulating *miR-133a*, and it exerts protective effects by activating the AMPK signaling pathway to restore autophagy. Moreover, H_2_S inhibits JNK1 and *miR-30c* entry, activates mTOR and PI3K/SGK1/GSK3β signaling pathways to inhibit autophagy, and alleviates I/R injury. Additionally, H_2_S prevents pyroptosis by inhibiting NLRP3/caspase-1. Given the complex pathological processes involved in I/R injury, further research on novel mechanisms is essential. Significant advancements in our understanding of the molecular mechanisms of H_2_S in reducing I/R injury have laid the groundwork for its potential in preventing and treating I/R injury in medical practice.

Several novel H_2_S donors have been designed and developed. Targeted drug delivery systems effectively transport H_2_S donors to specific organs or tissues. For instance, the mitochondria-targeted AP39 is commonly used to treat myocardial I/R injury, while ATB-344, an H_2_S-releasing form of indomethacin, prevents and treats gastric damage from I/R injury. Longer-term studies are needed to determine whether H_2_S can alleviate organ damage caused by I/R injury in clinical settings.

In conclusion, H_2_S donors and related drugs hold significant therapeutic potential for I/R injury, but their clinical translation requires extensive interdisciplinary research.

## Figures and Tables

**Table 1:** H_2_S and I/R injury. ↓: downregulation, ↑: upregulation.

Classify of I/R	H_2_S Donors (Concentration)	Effects	Mechanisms	Reference
Myocardial I/R	NaHS (400 μg/kg)	Cardiomyocyte apoptosis ↓	CSE/H_2_S pathway ↑	[33]
		Oxidative damage and apoptosis in cardiomyocytes ↓	CaR-CSE/H_2_S pathway ↑	[33]
		Myocardial infarction and ventricular dysfunction ↓	CSE expression ↑	[34]
		Cell apoptosis and oxidative stress ↓	CSE and H_2_S ↑	[35]
		Myocardial infarction ↓	H_2_S levels ↑	[36]
		Cardiomyocyte apoptosis ↓	H_2_S levels ↑	[36]
		Myocardial infarction area and restore left ventricular function ↓	cGMP/PKG pathway and H_2_S levels ↑	[37]
		Myocardial injury and enhancing cardiac function in rats ↓	Oprm1/miR-30b-5p/CSE/H_2_S axis ↑	[38]
		Cardiomyocyte apoptosis ↓	Oprm1/miR-30b-5p/CSE/H_2_S axis ↑	[38]
	NaHS (40 μM)	Myocardial injury ↓	CSE expression ↑	[39]
	NaHS (3 mg/kg)	Cardiomyocyte apoptosis ↓	K_ATP_ pathway ↑, MAPK and NF-κB pathway ↓	[40]
	NaHS (40 μM)	Heart function ↑	K_ATP_ pathway ↑	[41]
	NaHS (1.4, 2.8, 14 μM/kg)	Myocardial infarction area and restore left ventricular function ↓	LDH, CK, CK-MB ↓	[42]
	NaHS (100–400 μM)	Cardiomyocyte apoptosis ↓	GRP78, CHOP, eIF2α ↓	[42]
	H_2_S (50–200 μM)	Cardiomyocyte apoptosis and ER/SR stress ↓	miR-133a expression ↑	[51]
	ADT (50 mg/kg)	Myocardial infarction area and restore autophagy ↓	AMPK pathway ↑	[43]
	Zofenopril (10 μg/kg)	Myocardial infarction area ↓	H_2_S and NO ↑	[15]
	DATS	Myocardial infarction area ↓	eNOS/NO ↑	[48]
	NaHS (100 μg/kg)	Myocardial infarction area ↓	cGMP/PKG/PLN ↑	[49]
	NaHS (14, 28 μM/kg)	Myocardial infarction and ventricular dysfunction ↓	c-Fos expression ↓	[50]
	Na_2_S (100 μg/kg)	Myocardial infarction and ventricular dysfunction ↓	CK-MB and FABP expression ↓	[52]
	NaHS (30 μM/kg)	Cardiomyocyte apoptosis ↓	miR-1 expression ↓	[53]
	NaHS (30 μM)	Cardiomyocyte apoptosis ↓	miR-1 expression ↓	[53]
	NaHS (10 μM)	Myocardial infarction area ↓	Sirt1/PGC1α pathway ↑	[54]
	NaHS (10 μM)	Myocardial infarction area and restore left ventricular function ↓	JAK2/STAT3 pathway ↑	[55]
	GYY4137 (12.5, 25, 50 mg/kg)	Oxidative damage and apoptosis in cardiomyocytes ↓	MAPK pathway ↓	[56]
	NaHS	Cardiomyocyte and mitochondrial membrane damage ↓	JNK pathway ↓	[57]
	NaHS (1, 10, 20 μM)	Myocardial infarction area and restore left ventricular function ↓	LDH, CK ↓	[58]
	Na_2_S (50 μg/kg)	Myocardial infarction area and restore left ventricular function ↓	mitochondrial dysfunction ↓	[59]
	NaHS (50 μM)	Oxidative damage ↓	mitochondrial complex IV expression ↓	[60]
	NaHS (0.122–250 μM)	Cardiomyocyte apoptosis ↓	CK2 expression ↓	[61]
	NaHS (30 μM/kg)	Cardiomyocyte apoptosis ↓	GSK-3β (Ser9) expression ↑	[62]
	NaHS (30 μM)	Cardiomyocyte apoptosis ↓	GSK-3β (Ser9) expression ↑	[62]
	NaHS (50 μM)	Myocardial infarction area ↓	AKT pathway ↑	[63]
	NaHS (50 μM)	Cardiomyocyte apoptosis ↓	AKT pathway ↑	[63]
	NaHS (30–100 μM/Kg)	Oxidative damage ↓	mTOR pathway ↑	[64]
	NaHS (10–100 μM)	Oxidative damage ↓	mTOR pathway ↑	[64]
	NaHS (30 μM)	Autophagy in cells ↓	PI3K/SGK1/GSK3β pathway ↑	[65]
	DATS (200 μg/kg)	Myocardial infarction area and restore left ventricular function ↓	eNOS/NO ↑	[66]
	DATS-MSN (2, 4 mg/kg)	Cardiomyocyte apoptosis and oxidative damage ↓		[13]
	DATS-MSN (2.2 μM)	Myocardial infarction area and restore left ventricular function ↓	TLR4/NLRP3 pathway ↓	[26]
	Na_2_S (1 μM/kg), GYY4137 (25 μM/kg), AP39 (0.25 μM/kg)	Myocardial infarction area and restore left ventricular function ↓	H_2_S and NO levels ↑	[70]
	NaHS (10 μM)	Myocardial tissue injury, cardiomyocyte apoptosis ↓ and cardiac function ↑	PI3K/Akt/GSK-3β pathway ↑	[71]
	NaHS (10 μM)	Myocardial tissue injury, cardiomyocyte apoptosis ↑ and cardiac function ↑	AMPK/mTOR pathway ↑	[72]
	NaHS (100 μM)	Cardiomyocyte apoptosis and oxidative damage ↓	HB-EGF/EGFR ↑	[73]
Cerebral I/R	NaHS (1, 5 mg/kg)	Cerebral oedema and cell apoptosis ↑		[74]
	NaHS (50 μM/kg)	Neuronal pyroptosis and inflammation ↓	IL-1β, IL-18, NLRP3/caspase-1/GSDMD pathway ↓	[75]
	H_2_S (40 ppm)	Cell apoptosis ↓	HSP70 expression ↑, PI3K/Akt/Nrf2 pathway ↓	[76]
	GYY4137 (5 mM)	Cerebral infarct size and cell apoptosis ↑	p38 MAPK, ERK1/2 and JNK pathway ↓	[77]
	NaHS (4.8 mg/kg)	Restore neural function	RhoA/ROCK2 pathway ↓	[78]
	NaHS (0.2, 0.4 mM/kg)	Oxidative stress, inflammation and cell apoptosis ↓		[79]
	NaHS (0.1–10 mM/kg)	MCA relaxation ↓	K_ATP_ pathway ↑	[80]
		Post-ischemic inflammation ↓	CSE/H_2_S pathway ↑	[81]
	NaHS (1.25, 2.5, 5 mg/kg)	Cerebral infarct size and cell apoptosis ↓	MDA expression ↓, SOD and GSH-Px expression ↑	[82]
	NaHS (0–250 μM)	Cell apoptosis ↓	PARP-1/AIF pathway ↓	[83]
	NaHS (1, 2, 4, 8, 16 mg/kg)	Infarct volume and neurological deficits ↓	LC3-II expression ↓, p62 expression ↑	[84]
	NaHS (1, 2, 4 mg/kg)	Infarct volume ↓	LC3-II expression ↓, p62 expression ↑	[85]
	NaHS (200 μM)	Autophagy ↓	LC3-II expression ↓, p62 expression ↑	[85]
	NaHS (5.6 mg/kg)	Infarct volume and neurological deficits ↓	LDH, capase 3, LC3-II ↓, p62 ↑	[86]
	NaHS (100 μM)	Cell apoptosis ↓	LC3-II expression ↓, p62 expression ↑	[86]
	NaHS (12.5–200 μM)	Diastolic blood vessels	CSE, 3-MST, H_2_S ↑, RhoA-ROCK ↓	[87]
	NaHS (50–200 μM)	Diastolic blood vessels	RhoA-ROCK ↓	[88]
	H_2_S (40, 80 ppm)	Infarct volume and neurological deficits ↓	PKC expression ↑, AQP4 expression ↓	[89]
	Na_2_S (25–100 mM)	Cell death ↓	SIRT6 expression ↑	[90]
Hepatic I/R	NaHS (20 μM/kg)	Liver damage ↓	Nrf2 expression ↑, NQO1, GST, HO-1, miR-34a expression ↓	[91]
	NaHS (14, 28 μM/kg)	Liver damage ↓	JNK1 pathway ↓	[92]
	NaHS (1, 3, 5, 7, 9 μM)	Cell apoptosis ↓	BCl2 ↑, Bax, JNK1 pathway ↓	[92]
	GYY4137 (133 μM/kg)	Liver damage and cell apoptosis ↓	AKT pathway and miR-21 expression ↑	[93]
	NaHS (20 μM)	Cell apoptosis ↓	AKT pathway and miR-21 expression ↑	[93]
	H_2_S-Exo	Liver function ↑		[94]
	NaHS (56 μM/kg)	Liver damage and cell apoptosis ↓	ATF6, PERK, GRP78, TRAF2, CHOP ↓	[95]
	NaHS (30 μM)	Liver damage and inflammation ↓	SPHK1/S1P pathway ↓	[96]
	NaHS (12.5, 25, 50 μM/kg)	The mitochondrial and hepatocellular injury ↓	Akt-GSK-3β pathway ↑	[97]
Renal I/R	GYY4137 (0.25 mg/kg)	Kidney function ↑	miR-21 expression ↓	[98]
	NaHS (500 μg/kg)	Kidney injury ↓ and renal function ↑		[99]
		Kidney injury ↓	CSE promoter methylation ↓, H_2_S ↑	[100]
		Kidney injury ↓ and renal function ↑	CSE/H_2_S pathway ↑	[101]
	Na_2_S	Kidney injury ↓	IL-6, IL-1β, nitrite, nitrate ↓	[102]
	NaHS (150 μM)	Liver damage and cell apoptosis ↓	HSP70, HO-1, HSP27 expression ↑	[103]
	NaHS (500 μg/kg)	Kidney injury ↓	NLRP3/Caspase-1 pathway ↓	[104]
	NaHS (100 μM)	Liver damage and cell apoptosis ↓	NLRP3/Caspase-1 pathway ↓	[104]
Gastric I/R	NaHS (160, 320, 640 mg/kg)	Gastric mucosal damage ↓	CSE expression ↑	[105]
	ATB-344 (7 mg/kg)	Gastric mucosal damage ↓	Anti-inflammation	[16]
	NaHS (30–300 μM)	Gastric mucosal damage ↓	Keap1 s-sulfhydration, MAPK pathway ↑, NF-KB pathway ↓	[106]
	NaHS, GYY4137	Gastric mucosal damage ↓	SOD-2, GPx-1 expression ↑	[107]
	AP39	Gastric mucosal damage ↓	HIF-1α, GPx-1, ATP production, mitochondria complex I, complex III, GST-α, p-AKT and p-mTOR ↑, 8-OHG, complex IV, TIMP1, IL-1β, IL-1R2, iNOS, p-NF-κB, p-ERK ↓	[108]
Intestinal I/R	NaHS (7, 14 μM/kg)	Intestine injury ↓	SOD and GSH-Px ↑, MDA ↓	[109]
	NaHS (2 μM/kg)	Intestine injury ↓	eNOS/NO ↑	[110]
		Intestine oxidative injury ↓	CSE/H_2_S ↑, iNOS/NO ↓	[111]
	NaHS (14 μM/kg)	Intestine oxidative injury ↓	BK pathway ↓	[112]
	GYY4137 (50 mg/kg)	Intestinal mucosal injury ↓	IL-6, IP-10, MIP-2 ↓	[110]
	NaHS (0.5 mg/kg)	Intestine oxidative injury ↓	HIF-1α expression ↑	[113]
Lung I/R	H_2_S (50, 100 μM)	Lung oxidative injury ↓	CSE/H_2_S pathway ↑	[114]
	NaHS (0.78 mg/kg)	Lung injury ↓	AQP1/AQP5 ↑, TLR4/Myd88/NFκB ↓	[115]
Spinal cord I/R	NaHS (30 μM/kg)	Spinal cord infarct zone ↓ and hind motor function ↑	miR-30c expression ↓	[116]
	NaSH (10, 100, 200 μM)	Cellular ischemic injury ↑	Beclin-1 and LC3II ↑, miR-30c ↓	[116]
Testicular I/R	NaHS (75 μM/kg)	Testicular torsion ↓	TNF-α, iNOS, MDA, NO, GSH, and SOD ↓	[108]
	NaHS (10 mg/kg)	Testicular torsion ↓	GSH ↑, MPO and AOPP ↓	[107]
Retinal I/R	H_2_S (80 ppm)	Neuronal injuries ↓	HSP-90α ↑, NF-κB, caspase-3, p-JNK ↓	[117]

## Abbreviations

HCAEC: Human Coronary Artery Endothelial Cell; H/R: hypoxia-reoxygenation; ER/SR: endoplasmic/sarcoplasmic reticulum; DATS: Diallyl trisulfide; MCA: middle cerebral artery; MDA: malondialdehyde; OGD/R: oxygen-glucose deprivation and re-oxygenation; HK2: Hexokinase 2.
